# On the Influence of Some of the Diseases of the Body on the Mind

**Published:** 1831

**Authors:** 


### XXXI.

### ON THE INFLUENCE OF SOME OF THE DISEASES OF THE BODY ON THE MIND. By Sir HENRY HALFORD, Bt. M. D. &c. &c.

WE did not receive the little volume in which the above interesting paper is contained,* until after the Review department of this number was closed. We can only therefore notice one or two of the subjects in our Periscope of the present quarter. This is of less consequence, as the essays of the eminent President are quite unconnected by any link, except the place where they were read. They will therefore form separate articles of analysis, without any injury to the volume in which they are recorded. We have selected the fourth essay in the work for our present notice, because one of the most eloquent, and certainly not the least interesting, in the whole collection.

Sir George Baker, among others, has exercised his powers on the influence of some of the passions of the mind on the functions of the body. The object of Sir Henry is to furnish a counterpart of that picture—namely, the effects of bodily diseases on the mind. These effects are more obvious to the physician than to any other class of society—and they vary according to the different parts of the body in which the diseases or disorders have their seats.

"For what can be more in contrast with each other, in their influence upon the mental powers, than an indigestion and a slight inflammation of the brain? A disorder in the digestive organs lays a weight upon the mind.

> 'Corpus onustum

> Hesternis vitiis animum quoque prægravat una,

> Atque affigit humo divinæ particulam auræ.'

> *Horace.*

It renders a man irresolute, infirm of purpose, and both indisposed and unequal to enterprise of any kind. Whilst a slight inflammation of the brain gives a sharpness* to his faculties, inspires spirit, quickens ambition, and leads him to believe, like Hotspur, that he can—

'Pluck bright honour from the pale-faced moon"

Perhaps if *excitement* were substituted for *inflammation* in the foregoing eloquent passage, it would be more generally applicable. In maniacs, for

---

* Essays and Orations read and delivered at the Royal College of Physicians. By Sir Henry Halford, 8vo. pp. 192, 1831.

* 'Multa enim e corpore existunt, quæ acuant mentem, multa quæ obtundant.'—Cic. Tusc. Lib. i. 33.

example, where these bright visions of the fancy go on for years, and after all, leave no traces of their existence in the brain, we cannot but conclude that it is irritation or excitement, rather than inflammation of the sensorium which keeps up the vivid trains of thought.

Of apoplexy our learned author professes to say little. Of the paralyses, the sequels of apoplexy, he presents us with the following sentiment.

"But the sequel of apoplexy is PALSY; and when that has supervened, and the frame has been dismembered, then, indeed, happy is the patient whose mind shall have been disciplined when in health, and whose moral habits shall have been well regulated by reason and by good principles before he was taken ill; for, otherwise, as all the passions are let loose by the malady, (as is the case in many instances, at least, in this disease,) whilst the controlling power is enfeebled, an irritability succeeds which makes life intolerable to the sick man himself, and to all around him. The tenderest offices, administered with the most prudent attention and care, fail to conciliate; and he indulges his anger, and dissolves into tears alternately, alike without reason, until at length another apoplectic blow deprives him of life.

By this distemper the great talents of Marlborough were confounded in the latter years of his life, and his powerful mind impaired. By this also was extinguished the spirit of the celebrated Dean Swift:—

'From Marlbro's eyes the tears of dotage flow,
And Swift expires a driveller and a show.'"

In respect to epilepsy, it is a merciful dispensation of Providence that, however frighful the attack may appear to others, the victim himself is unconscious of the seizure. But it is too well known that repeated fits debilitate the mental faculties, and even lead to fatuity. Sir Henry is of opinion that Julius Cæsar's epilepsy was symptomatic of some derangement in the alimentary canal; as it did not seem to impair his faculties.

The next contrast which our author draws is that which the young hectic female presents as compared with that disordered state which sometimes occurs after the cessation of the catamenia.

"The subject of such an indisposition has probably grown more corpulent; she sits in an indolent posture, looks gloomy, hardly speaks at all, and we learn from her attendants that she lives under a constant apprehension that some fancied evil is about to befal her. She is suspicious, undecided in all her movements, and manifests symptoms which *differ in degree only* from melancholy mania.

The pathologist will look, perhaps, to the different state of the circulation of the blood in these two females for the difference of their animal spirits; and will conjecture that the blood was more oxygenated in the younger one, by a more rapid circulation through the lungs, whereby the brain was unusually stimulated; whereas, in the elder person, there was a stagnation in the liver, giving rise to hypochondriasm, in consequence of the more gorged, plethoric state of the ventral and hæmorrhoidal veins determined to that organ, since the sexual evacuation had ceased."

The agonizing paroxysms of suffocation occasioned by organic diseases of the heart are next sketched in the energetic language of the author. In the intervals between the attacks (of angina pectoris) the mind is often cheerful and buoyant, and the patient indulges sanguine hopes.

"Hence, the subjects of such painful disorders are commonly less dejected than those who suffer only from a derangement of the stomach. Whether it be that Providence has specially allotted a certain alacrity of spirit and cheer of mind to the victims of this disease of the main-spring of life, as an alleviation of their sufferings, or whether this may be referred to the general principle which Dr. Paley has stated with respect to pain, 'that its pauses and intermissions become positive pleasures; that it has the power of shedding a satisfaction over the intervals of ease which few enjoyments exceed.' This amiable philosopher adds, that 'the spirits of sick men do not sink in proportion to the acuteness of their sufferings, but rather appear to be roused and supported, not by pain, but by the high degree of comfort which they derive from its cessation, or even its subsidency, whenever that occurs, and which they taste with a relish that diffuses some portion of mental complacency over the whole of that mixed state of sensations in which disease has placed them.' "

The foregoing observations evidently apply only to what has been vaguely termed sternalgia, angina pectoris, syncope anginosa, and other learned names. In organic affections of the heart generally, we have found a very considerable depression of spirits prevail—often to a most distressing extent. And no wonder! For in such diseases, the circulation is never entirely free from embarrassment, and the respiratory function, on which life so imminently depends, is seldom free.

"That pain alone does not affect the faculties, is manifested in that most excruciating of all disorders, tic douloureux. Nay, where pain is conjoined with other symptoms, calculated to subdue the stoutest heart, as in the progress of a fatal iliac passion, it does no violence to the senses. In this dreadful disease, in which hiccup, unquenchable thirst, incessant vomiting, unspeakable inquietude, prevail for six or seven successive days and nights before the scene of misery be closed, yet does the patient maintain his mental powers; and, spite of the constant disappointment of his expectations of being relieved by the operation of his medicine, does he exercise his judgment and keep up his hopes."

Sir Henry adverts to the unmanly, or at least unchristian practice of the ancient Romans, who considered themselves at liberty to terminate their own existence when their diseases were painful, or their physicians pronounced them to be incurable.

"Their creed admitted an independent exercise of their free-will and pleasure in the disposal of their lives:—

Ipse Deus, simul atque volam me solvet—

Moriar. *Mors* ultima linea rerum est."

Of the great number which it has been our author's painful duty to attend in the last hours of their lives, he has sometimes felt surprised that so few have appeared reluctant to go to the "undiscovered country from whose bourne no traveller returns." Many, he thinks, may have manifested this willingness to die, from an impatience of suffering, or from the passive indifference which is sometimes the result of debility and bodily exhaustion; but, "he has seen those who have arrived at a fearless contemplation of the future, from faith in the doctrine which our religion teaches." Some indeed have clung to life anxiously—painfully; but they did not appear to be so much influenced by a love of life, for its own sake, as by the distressing prospect of leaving children, dependent on them, to the mercy of the world.

The following piece of medical ethics, from a man of such experience and tact as Sir Henry Halford, is deserving of record in the author's own words.

"And here you will forgive me, perhaps, if I presume to state what appears to me to be the conduct proper to be observed by a physician in withholding, or making his patient acquainted with, his opinion of the probable issue of a malady manifesting mortal symptoms. I own I think it my first duty to protract his life by all practicable means, and to interpose myself between him and every thing which may possibly aggravate his danger. And unless I shall have found him averse from doing what was necessary in aid of my remedies, from a want of a proper sense of his perilous situation, I forbear to step out of the bounds of my province in order to offer any advice which is not necessary to promote his cure. At the same time, I think it indispensable to let his friends know the danger of his case the instant I discover it. An arrangement of his worldly affairs, in which the comfort or unhappiness of those who are to come after him is involved, may be necessary; and a suggestion of his danger, by which the accomplishment of this object is to be obtained, naturally induces a contemplation of his more important spiritual concerns, a careful review of his past life, and such sincere sorrow and contrition for what he has done amiss, as justifies our humble hope of his pardon and acceptance hereafter. If friends can do their good offices at a proper time, and under the suggestions of the physician, it is far better that they should undertake them than the medical adviser. They do so without destroying his hopes, for the patient will still believe that he has an appeal to his physician beyond their fears; whereas, if the physician lay open his danger to him, however delicately he may do this, he runs a risk of appearing to pronounce a sentence of condemnation to death, against which there is no appeal—*no hope;* and, *on that account,* what is most awful to think of, perhaps the sick man's repentance may be less available.

But friends may be absent, and nobody near the patient in his extremity, of sufficient influence or pretension to inform him of his dangerous condition. And surely it is lamentable to think that any human being should leave the world unprepared to meet his Creator and Judge, 'with all his crimes broad blown!' Rather than so, I have departed from my strict professional duty, and have done that which I would have done by myself, and have apprized my patient of the great change he was about to undergo."

These considerations bring our author naturally to a defence of his conduct in cautiously wording the bulletins of his late Majesty's last illness—a vindication which is unnecessary, as far as the medical profession is concerned, and to which we did ample justice in this journal, at the time when the melancholy event took place. The following particulars, however, are deserving of notice, and with them we shall conclude this article.

"In the case of his late Majesty, the King's Government and the Royal Family were apprized, as early as the 27th of April,* (I hold in my hand the original letters which gave the information to the Prime Minister), that his Majesty's disease was seated in his heart, and that an effusion of water into the chest was soon to be expected. It was not, however, until the latter end of May—when his Majesty was so discouraged by repeated attacks in the embarrassment in his breathing, as to desire me to explain to him the nature of his complaint, and to give him my candid opinion of its probable termination—that the opportunity occurred of acknowledging to his Majesty the extent of my fears for his safety.

This communication was not necessary to suggest to the King the propriety of religious offices, for his Majesty had used them daily. But it determined him, perhaps, to appoint an early day to receive the Sacrament. He did receive it with every appearance of the most fervent piety and devotion, and acknowledged to me repeatedly afwards, that it had given him great consolation—true comfort.

After this, when 'he had set his house in order,' I though myself at liberty to interpret every new symptom as it arose in as favourable a light as I could, for his Majesty's satisfaction; and we were enabled thereby to rally his spirits in the intervals of his frightful attacks, to maintain his confidence in his medical resources, and to spare him the pain of contemplating approaching death, until a few minutes before his Majesty expired.

Lord Bacon,* one of the wisest men who has lived, encourages physicians to make it a part of their art to smooth the bed of death, and to render the departure from life easy, placid, and gentle.

This doctrine, so accordant with the best principles of our nature, commended not only by the wisdom of this consummate philosopher, but also by the experience of one of the most judicious and conscientious physicians of modern times, the late Dr. Heberden, was practised with such happy success in the case of our late lamented sovereign, that at the close of his painful disease 'non

* "His Majesty died on the 25th of June."

* "See chap. ii., lib. 4, 'De Augmentis Scientiarum.'

tam mori videretur (as was said of a Roman Emperor) quam dulci et alto sopore excipi.' "

---

XXXII.

ON POISONED CONFECTIONARY. By W.B. O'SHAUGHNESSY, M.D.

